# 3D Printing and Guided Endodontics: The Future of Precision Access Cavity Design

**DOI:** 10.7759/cureus.97734

**Published:** 2025-11-25

**Authors:** Samsul A Choudhury, Sudarshan Pal, Sanchari Sen, Shubham K Srivastava, Chinmoy Sikdar

**Affiliations:** 1 Conservative Dentistry and Endodontics, Career Post Graduate Institute of Dental Sciences and Hospital, Lucknow, IND; 2 Prosthodontics, Sardar Patel Post Graduate Institute of Dental and Medical Sciences, Lucknow, IND

**Keywords:** computer-aided design, computer-aided manufacturing, cone-beam computed tomography, guided endodontic access, three-dimensional printing

## Abstract

The evolution of endodontic practice is witnessing a paradigm shift toward precision and minimally invasive interventions. Guided endodontics, empowered by advancements in three-dimensional (3D) printing and digital imaging, has transformed the way access cavities are planned and executed. Conventional access approaches can sometimes lead to excessive removal of dentin, prompting interest in more conservative techniques. With the integration of cone-beam computed tomography (CBCT), intraoral scanning, and computer-aided design/computer-aided manufacturing (CAD/CAM), clinicians can now visualize internal root anatomy preoperatively and fabricate patient-specific guides that direct access burs with high accuracy. This study explores the technological foundation, clinical applications, and limitations of 3D-printed guided endodontic systems, emphasizing their role in preserving critical tooth structure and improving treatment predictability. It also discusses the future potential of artificial intelligence (AI) and real-time navigation systems in enhancing clinical precision and accessibility.

## Editorial

Traditional endodontic access cavity design relies heavily on the clinician’s experience, tactile feedback, and radiographic interpretation. Although these principles have served as the foundation of endodontic therapy, they are inherently limited by anatomical variability and operator subjectivity. The modern philosophy of minimally invasive endodontics (MIE) prioritizes conservation of pericervical dentin, a critical determinant of long-term tooth strength and fracture resistance. However, conservative access can increase the risk of missed canals or incomplete debridement. To reconcile these competing objectives, guided endodontics integrates advanced digital imaging and additive manufacturing to create customized templates that direct the bur precisely to the target canal [1].

The rationale for guided endodontics

The primary objective of endodontic therapy is to eliminate pulpal pathology while preserving sound tooth structure. However, calcifications, restorations, or distorted anatomy can render conventional access unpredictable. Guided endodontics translates virtual planning into clinical reality through a digital workflow combining cone-beam computed tomography (CBCT) data and intraoral scanning [1].

CBCT imaging provides a volumetric view of canal trajectories, while surface scans generate accurate digital replicas of the coronal tooth anatomy. When superimposed, these datasets allow clinicians to plan an optimized access pathway with micrometer-level precision. The digital plan is then converted into a three-dimensional (3D)-printed guide that fits the tooth, dictating the angulation and depth of the bur. This approach ensures reproducibility across operators and minimizes reliance on subjective judgment [2].

Studies have consistently demonstrated that guided access cavities produce significantly higher accuracy and lower dentin loss compared to freehand techniques [1,2]. The method is especially beneficial for anterior teeth with pulp canal obliteration (PCO), where visual and tactile cues are unreliable. Moreover, guided approaches substantially reduce the risk of iatrogenic complications such as perforations or missed canals [3]. In this context, bur selection becomes a pivotal factor influencing accuracy and clinical success. Guided endodontics typically employs long-shank, small-diameter burs, ranging from approximately 0.5-1.3 mm, to ensure compatibility with the guiding sleeve and to maintain a conservative yet precise access path. These burs, often carbide or diamond-coated, are designed to minimize flex and vibration during drilling, thereby supporting the stability of the static guide. In cases with severe calcification, initial penetration using these guided burs can be complemented with ultrasonic tips or micro-drills to refine the access while maintaining directional control. Proper matching of bur dimensions with the guide sleeve is essential to prevent deviation and enhance the overall predictability of the procedure.

The role of 3D printing in workflow integration

Additive manufacturing forms the technological backbone of guided endodontics. The workflow begins with CBCT and intraoral data acquisition, followed by digital planning of the ideal access trajectory. Effective virtual planning requires several key requisites as follows: high-resolution CBCT scans with minimal scatter, accurate surface scans for reliable superimposition, and proper calibration of software tools to ensure precise alignment of datasets. The clinician must also verify anatomical landmarks, evaluate remaining tooth structure, and confirm adequate interocclusal clearance to accommodate the guide sleeve and bur dimensions. Once these prerequisites are met, planning software enables virtual alignment of the access path, including the insertion angle, entry point, and target depth, ensuring a predictable and conservative access design. A guiding sleeve is incorporated to control the bur path, enhancing precision during access [4].

Using stereolithography (SLA), digital light processing (DLP), or PolyJet 3D printing technologies, the guides are fabricated from biocompatible resin materials that provide excellent dimensional accuracy, typically within 0.2 mm of the planned trajectory [1]. PolyJet printing, in particular, offers smoother surface detail and multi-material capabilities, improving guide fit and optical clarity during intraoral placement. The patient-specific design enhances stability, while rapid prototyping enables same-day fabrication in clinics equipped with in-house printers. This integration streamlines workflow efficiency and supports the increasing adoption of guided endodontics in contemporary clinical settings [4].

Clinical applications

Guided endodontics has demonstrated success in various challenging clinical scenarios. Its most validated application lies in the management of calcified or obliterated canals. In such cases, conventional probing carries a high risk of perforation or excessive dentin loss. By directing the bur along a digitally preplanned trajectory, 3D-printed templates offer a conservative and predictable solution for negotiating these complex canals [3]. Importantly, this approach aligns closely with the principles of minimally invasive endodontics, enabling the preservation of pericervical dentin (PCD) - a structural zone critical for long-term tooth strength and resistance to fracture.

The technique has also proven particularly valuable in retreatment cases, where existing restorations or altered anatomy often obscure canal orifices. In these situations, guided re-entry minimizes unnecessary removal of restorative material and significantly reduces the risk of iatrogenic complications, such as perforation, ledging, or deviation from the canal path. Moreover, guided systems facilitate predictable localization of accessory canals, such as the mesiobuccal 2 (MB2) canal in maxillary molars, which remains one of the most frequently overlooked structures in traditional practice [1,2].

Recent literature has expanded the scope of guided endodontic principles to periradicular surgery, including guided apicoectomy and retrograde preparation [3]. Such developments highlight the increasing versatility of this technology across both nonsurgical and surgical endodontic procedures.

Beyond the established clinical indications, several practical considerations further refine the applicability of guided endodontics in daily practice. The materials used in guide fabrication play a crucial role in accuracy and safety; most templates are 3D-printed using biocompatible light-cured resins, while emerging autoclavable polymer formulations now allow more reliable sterilization without compromising dimensional stability. The fabrication workflow, from CBCT acquisition and intraoral scanning to CAD-based planning, guide design, and additive manufacturing, enables a streamlined and highly reproducible process that translates virtual planning into precise clinical execution. In terms of clinical use, guides demonstrate their greatest value in retreatment and calcified-canal scenarios, where altered anatomy, restorations, or canal obliterations make freehand access unpredictable. However, their use in initial endodontic therapy may be limited when extensive caries or significant coronal destruction prevents proper seating or stabilization of the template, reducing accuracy. Understanding these material, workflow, and case-selection considerations is essential for integrating guided endodontics effectively and safely into contemporary practice.

Limitations and current challenges

Despite its benefits, guided endodontics faces notable challenges. High equipment and software costs limit its widespread adoption in routine practice [4]. The workflow also demands technical expertise in digital planning and familiarity with computer-aided design (CAD) software, along with careful attention to data privacy and security when AI-driven applications are incorporated into the planning process. While AI enhances accuracy, it cannot replace the clinician’s judgment in complex clinical scenarios that require nuanced interpretation.

Physical constraints, such as limited interocclusal space in posterior regions, can restrict guide placement. Inaccurate registration between CBCT and surface data or improper guide seating may result in cumulative deviations, leading to canal misalignment [3,4]. Furthermore, guided templates are patient-specific and designed exclusively for single use; reusing a guide is neither practical nor advisable, as cold-sterilization protocols required for most non-autoclavable resin materials may compromise dimensional stability over time [4].

The static nature of these guides precludes real-time adjustments once fabrication is complete. Consequently, guided techniques are currently best suited for anterior and single-rooted teeth, while posterior multi-rooted cases may still pose challenges [1,5].

Future directions and technological integration

The next phase of innovation in guided endodontics will likely arise from the integration of dynamic navigation systems, artificial intelligence (AI)-assisted planning, and robotic micro-drilling. Dynamic navigation, already well established in implantology, provides live visual feedback during drilling, merging the accuracy of guidance with real-time adaptability [5]. AI-based algorithms are now being developed to automatically identify canal trajectories and design access paths from CBCT datasets, reducing operator variability and enhancing planning precision. Notably, the combination of convolutional neural networks (CNNs) and artificial neural networks (ANNs) has driven significant progress in image recognition tasks, enabling more reliable detection of calcified canals and complex anatomical structures. As these systems evolve, their convergence with chairside 3D printing may allow automated guide fabrication and on-demand planning within a single appointment.

Material science is also advancing toward autoclavable, transparent, and flexible polymers, improving clinical safety, visualization, and usability [5]. As technological costs continue to decline and accessibility increases, guided endodontics is poised to evolve from a specialized innovation into a mainstream modality in contemporary practice.

Conclusion

3D printing and guided endodontics represent a transformative advancement in precision dentistry. By combining digital imaging, computer-assisted planning, and additive manufacturing, clinicians can perform access cavity preparations with unprecedented accuracy and minimal invasiveness. Although cost and technical barriers persist, rapid technological evolution promises to make these techniques more accessible and standardized. The transition from freehand to guided endodontics mirrors the digital transformation seen in implantology - signifying a future where accuracy, preservation, and predictability define endodontic excellence. Continued clinical validation through standardized protocols and longitudinal trials will ultimately cement guided endodontics as an integral component of modern dental practice.
